# Truncal Acne: An Overview

**DOI:** 10.3390/jcm11133660

**Published:** 2022-06-24

**Authors:** Yu Ri Woo, Hei Sung Kim

**Affiliations:** Department of Dermatology, Incheon St. Mary’s Hospital, The Catholic University of Korea, 222 Banpo-daero, Seocho-gu, Seoul 06591, Korea; w1206@naver.com

**Keywords:** acne, face, truncal acne

## Abstract

Acne is a relatively common disease of the pilosebaceous units. Many aspects of facial acne have been studied. However, there is limited evidence regarding truncal acne. Truncal acne is also observed in a significant number of patients, but it is often ignored by patients and clinicians. Although the pathogenesis of facial and trunk acne is considered to be similar, the characteristics of the skin on the trunk and face are thought to be different. As truncal acne can cause scars on large areas of the body and adversely affect the quality of life of patients, more attention should be given to patients with truncal acne. Although only a few studies have been published to date, the epidemiology, etiology, severity assessment tool, assessments of the quality of life, and new treatments targeting truncal acne are currently being studied. Therefore, in this review, the latest knowledge on truncal acne will be discussed.

## 1. Introduction

Acne vulgaris is a common cutaneous disorder of the pilosebaceous unit with a high prevalence in the general population. A global burden of disease study in 2010 identified acne vulgaris as the eighth most common skin disease, with an estimated global prevalence of 9.38% [1]. A recent systematic review also estimated the prevalence of acne to range from 20% to 95% [2]. Of note, the global lifetime prevalence of acne is estimated to be 70–85% [3]. Despite its high prevalence, truncal acne has been neglected by many clinicians and patients. Although there has been a paucity of evidence on the epidemiology, pathogenesis, clinical features, and treatment of truncal acne, some recent studies are updating the evidence on truncal acne. Therefore, this review aimed to provide the evidence-based recent perspectives on truncal acne.

## 2. Epidemiology

The prevalence of truncal acne has not yet been well established to date. Previous studies report that about 48–52% of facial acne patients also have truncal acne [4,5,6]. In 2007, Del Rosso et al. [4] examined 696 patients aged 14 to 20 years with acne and found that 52.3% exhibited truncal involvement. Among them, 10.6% showed truncal acne scarring [4]. Isaacsson et al. [5] reported that 50% of Brazilian adolescents had acne on their chest or back. A large-scale international study of 2926 adult females found that 48.8% of the patients with facial acne also had truncal acne [6]. Recently, Dreno et al. reported that a family history of acne was associated with the extension of acne to the trunk [7].

With regards to sex, there is a slight male predominance of truncal acne (54%) over female (43%) [4]. Truncal acne is known to occur in adolescents as well as adults. Despite the results of the above-mentioned studies, few studies have studied only truncal acne, and additional research including large-scale and diverse ethnic groups is needed to identify the epidemiology of truncal acne.

## 3. Clinical Features of Truncal Acne

**Figure 1:**
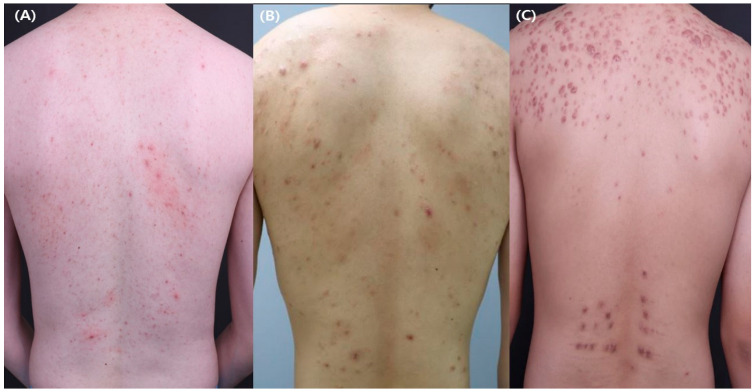
Clinical manifestations of truncal acne. (**A**,**B**) Truncal acne. (**C**) Truncal acne with severe scarring.

The clinical manifestations of truncal acne can range from noninflammatory comedones to inflammatory papules, pustules, and nodules located on the chest and/or back (Figure 1). When truncal acne is observed, it tends to be clustered near the midline of the trunk [8]. As the severe form of acne is usually accompanied by truncal involvement [9], truncal acne should always be considered by clinicians in cases of severe facial acne.

The most frequently involved anatomical site of truncal acne is the upper back (52%), followed by the upper chest (30%), lower back (22%), shoulders and upper extremities (16%), and neck (8%) [10]. A study conducted in East China also found that the back (25%) was the most frequently involved anatomical site, followed by the chest (16%), neck (7%), and arms (6%) [11]. A multicenter questionnaire-based study conducted in Korea found that the site most frequently affected with truncal acne in males was the back (19.4%), followed by the chest (18.1%) [12]. In females, the most frequently affected site was the back (13.9%), followed by the neck (12.7%) and chest (11.1%), which showed slight sex differences in the sites affected by acne [12]. Although there are some inconsistencies between the studies, most found that the back was the most frequently affected area in truncal acne. Scar formation from truncal acne is common and is mainly hypertrophic [9].

A severe form of acne, acne conglobata, usually presents with nodules, abscesses with sinus formation, and cysts on the chest, shoulders, back, and upper extremities [13]. As acne conglobata is characterized by suppurative severe inflammation, scar formation is common [13]. Acne fulminans is also known as acute febrile ulcerative acne and is the most severe type of acne [14]. It can be manifested with or without systemic symptoms and often heals with scarring [14]. The sudden appearance of tender inflammatory plaques with ulcers and hemorrhagic crusts on the chest and back, whilst sparing the neck, can be the characteristic signs of acne fulminans [14]. Nodules and polymorphous comedones are less frequently observed in acne fulminans than in acne vulgaris [14].

## 4. Pathophysiology of Truncal Acne

To date, the etiology of truncal acne is generally considered to be similar to that of facial acne. Follicular hyperkeratosis, the colonization of pilosebaceous units with *Cutibacterium acnes* (*C. acnes*), and inflammation are all considered major pathogenic factors in the development of facial and truncal acne [15,16]. Although facial and truncal acne have been considered to have shared pathogenesis, some differences between them have been reported. Kim et al. [17] found that sebum secretion was relatively lower in the trunk than in the face of patients with acne vulgaris. They suggested that hyperseborrhea might not play a major pathogenic role in the development of truncal acne [17].

Human hormones affect the biological functions of the pilosebaceous unit. The rise in androgen level during puberty is associated with the development of acne [18]. Among various hormones, receptors for androgens are also found in sebocytes and follicular keratinocytes [18]. Androgen can induce lipogenesis of the sebaceous gland, hyperproliferation of the follicular keratinocyte, proliferation of *C. acnes*, and follicular hypoxia, which could contribute to the development acne [18,19].

A variety of evidence has proved a link between the gut microbiota and acne. Previous studies found that patients with acne had dysbiosis of the gut microbiota [20,21,22]. However, to date, no study has analyzed the characteristics of gut microbiota in patients with truncal acne, and further studies are needed. In addition to the gut microbiota, alterations in the skin microbiota are also considered an important player in truncal acne. Dagnelie et al. [23] reported differences in the dominant bacterial families between facial acne and truncal acne. Colonization with *Enterococcaceae* was more frequently observed in truncal samples, whereas colonization with *Staphylococcaceae* and *Propionibacteriaceae* was frequently observed in facial samples [23]. Using the single-locus sequence typing method, Dagnelie et al. [24] found that phylotype IA1 was the most predominant type on the back of the acne patients compared to the healthy controls. Severe acne on the back was associated with a loss of *C. acnes* phylotype diversity [24]. Taken together, alterations in the composition of the skin bacterial microbiota might affect the inflammatory processes of the acne skin. In addition to the bacterial microbiota, the possible role of the fungal mycobiota in the development of truncal acne should be investigated further. Although little is known about the fungal mycobiota in truncal acne, a recent study found a negative interaction between *Malassezia globosa* and *C. acnes* among patients with facial acne [25]. Thus, we suppose that an interaction between the bacterial microbiota and fungal mycobiota, along with host factors, might be involved in the development of truncal acne, which remains to be elucidated in the future.

Endocrine abnormalities are also associated with acne [26]. Acne is a frequently observed clinical feature of polycystic ovarian syndrome (PCOS), congenital adrenal hyperplasia, Cushing syndrome, and androgen-secreting tumors [26,27]. Among patients with female acne, acne with hyperandrogenic signs was more strongly associated with truncal acne, longer disease duration, inappropriate diet, and PCOS than acne without hyperandrogenic signs [28].

As truncal acne is associated with post-acne scarring, some studies have investigated the mechanisms of scarring in truncal acne. A recent study found a difference in the profile of skin innate immunity in patients with acne by performing skin biopsies of the back [29]. The significant overexpression of pro-inflammatory markers such as Toll-like receptor-4 and interleukin-2 was observed both in the normal-appearing skin and inflammatory skin of acne patients with atrophic scars compared to acne patients without atrophic scars [29]. The authors suggested that the degree of activation of innate immunity was associated with the levels of acne inflammation and further scar formation [29]. 

Hypertrophic post-acne scarring was associated with the CC genotype of *MMP-2* [30]. Among patients with hypertrophic acne scars, 95.8% were shown to have the CC genotype of *MMP-2* [30]. Hypertrophic acne scarring patients with the CC genotype of *MMP-2* showed a 7.8-times increased risk of developing hypertrophic acne scars than acne patients without scar formation [30].

Although the etiology of truncal acne is generally considered to be similar to that of facial acne, there are differences between the skin on the face and the trunk in thickness, pH, and the distribution of sebaceous glands. Truncal skin is also more vulnerable to mechanical stimuli such as sweat, oils, pressure, friction, and occlusion than facial acne [31]. We hypothesize that these factors might be associated with the severity of truncal acne. Further large-scale clinical studies should be conducted to elucidate the relationship between these factors.

## 5. Assessment Tool for Truncal Acne

Previously, a tool for assessing the severity of truncal acne was used along with a tool for evaluating facial acne. 

After Pillsbury et al. [32] proposed a tool for assessing truncal acne, Burke et al. [33] introduced the Leeds technique in 1984, which uses two simple scoring systems for evaluating the acne severity. In 1998, O’Brian et al. [34] proposed the Leeds Revised Acne Grading (LRAG) system based on the photographic templates for acne grading of the face, chest, and back. Tan et al. [35] introduced the comprehensive acne severity scale (CLASS) by modifying the investigator’s global assessment of facial acne to evaluate truncal acne severity. The CLASS was strongly correlated with the Leeds grading system in a validation study [35]. In 2010, Tan et al. [36] developed a scale for assessing acne scar severity by developing a six-category global severity scale (SCAR-S) evaluating the severity of each region of the face, chest, and back.

Bernadis et al. [37] proposed a new acne global grading scale in 2021 for assessing acne severity by combining primary lesions and secondary changes. A very recent study provided the truncal acne severity scale (TRASS), which is very easy for dermatologists to use for assessing the severity of truncal acne. The TRASS evaluates the severity of truncal acne by utilizing a severity tool based on the global evaluation acne (GEA) and the Echelle de Cotation des Lesions d’Acne (ECLA) scales, and includes an assessment tool considering family history, clinical signs, and quality of life (QoL) [38]. In the TRASS, the xiphoid process is defined as the lower limit for the location of chest acne [38]. For back acne, the back is divided into two regions compromising the upper and lower backs, with the lower limit of the scapular defined as a line to separate them [38]. 

Despite the development of various assessment tools for truncal acne, the above-mentioned tools are mostly used as tools for clinical research (Table 1). In real-world clinical practice, the investigator’s global assessment (IGA), the Leeds visual severity scale, ECLA scales, and the comprehensive acne severity scale are widely used for assessing the severity of truncal acne [39]. Given the anatomical variations in acne, there are unmet needs to properly evaluate the severity of truncal acne in real-world clinical practice. Therefore, more attention should be paid to develop more practical assessment tools for truncal acne in the future.

## 6. Differential Diagnosis of Truncal Acne

A variety of papulopustular eruptions on the trunk should be differentiated from truncal acne.

Folliculitis is defined as an infection of the hair follicles by *Staphylococcus aureus* or other factors [40]. The clinical features of folliculitis are characterized by solitary or multiple inflammatory follicular erythematous papules and/or pustules without comedones on hair-bearing areas [40]. Pityrosporum folliculitis is the cutaneous disorder most frequently misdiagnosed as truncal acne. Clinically, pityrosporum folliculitis is manifested by pruritic monomorphic tiny follicular papules and pustules with or without perifollicular erythema on the upper portion of the trunk, neck, and upper arms [41]. Pityrosporum folliculitis can be differentiated from truncal acne by the absence of comedones, the lack of response to topical or oral antibiotics, and the presence of pruritic skin lesions [42].

In addition, acneiform eruptions, which are caused by several drugs including glucocorticoids, phenytoin, lithium, isoniazid, high-dose vitamin B complex, halogenated compounds, cyclosporine, epidermal growth factor receptor inhibitors, and BRAF inhibitors, are always considered when diagnosing truncal acne [43]. Usually, drug-induced acneiform eruptions present as diffuse monomorphic follicular papules on the trunk. Regarding steroid folliculitis, skin eruptions can generally be observed after two weeks of steroid use [43]. Unlike acne vulgaris, steroid folliculitis usually presents as inflammatory papules and pustules on the trunk, shoulders, and upper arms, with less involvement on the face. Comedones, cysts, and scarring are uncommonly observed, although post-inflammatory hyperpigmentation could be observed after improvement [43]. Recently, the term “bodybuilding acne” has been proposed for individuals who have been administered anabolic androgenic steroids such as methandrostenolone, nandrolone, testosterone, oxandrolone, stanozolol, or methenolone, which are usually taken by bodybuilders [18]. The clinical manifestations of bodybuilding acne vary from the occurrence of new-onset acne to the exacerbation of pre-existing acne, or the occurrence of acne conglobate or acne fulminans [18]. In addition to the above-mentioned medications, diverse drugs have recently been shown to cause acneiform eruptions. Therefore, for the correct diagnosis of drug-induced acneiform eruptions, it is necessary to properly evaluate the relationship between the drug intake history and the onset of the skin lesions through a detailed medical history [43].

## 7. Psychosocial Burden of Truncal Acne

The degree of the impact of acne on QoL is similar to that of other chronic general disorders such as diabetes, asthma, epilepsy, back pain, or arthritis [44]. Although the negative impact on QoL is correlated with the severity of acne, mild acne patients can also suffer from a significant negative impact on QoL, despite their mild signs [45].

To date, most previous studies determining the psychosocial burden of acne have been focused only on the impact of facial acne. As the face is the exposed area of the body, much attention is indeed paid to the facial regions. However, interest in truncal acne has increased recently, and studies on the effects of truncal acne on QoL are being conducted. A study by Papadopoulos et al. [46] found that the patients with facial and truncal acne had significantly lower self-esteem and body image than the controls. Compared to truncal acne patients, patients with facial acne exhibited lower self-esteem and body image [46]. A recent cross-sectional study identified that the negative impact on all health-related QoL was higher in patients with combined facial and truncal acne than in facial acne patients [47]. Among a variety of health-related QoL domains, having combined facial and truncal acne was associated with poorer emotional well-being, everyday life activities, participation in social activities and sports, and routine acne treatment than having facial acne alone [47].

The burden of truncal acne is obvious in many patients; however, it is thought that the degree of the impact of truncal acne could vary from patient to patient, and there is a difference in psychosocial burdens between patients with truncal acne and those with facial acne. As the truncal regions are usually exposed in summer, there might be a seasonal variation in the burden of truncal acne. The consensus of an expert panel suggested that the visual analog scale (VAS) could be a useful tool for assessing the factors associated with the impact of disease on patients, and thereby help to identify the treatment goal for each patient [39].

## 8. Management of Truncal Acne

In general, treatment-specific guidelines for acne have been established and updated in various countries [48,49,50,51]. However, the management guidelines for truncal acne are limited. In clinical practice, some clinicians are managing truncal acne based on the guidelines for facial acne. The recent consensus of an expert panel recommended managing truncal acne similar to facial acne [39]. Although the underlying pathogenic mechanisms between facial acne and truncal acne are similar, as there are some differences between the face and trunk, the management of truncal acne should be considered from a slightly different perspective. As the involved body surface area is broader in truncal acne than facial acne, and there is a difference in the characteristics of the skin between the trunk and face, these aspects should always be considered when choosing optimal treatment options for truncal acne. Although the clinical evidence for using topical and systemic agents for truncal acne is limited, some recent evidence confirmed the clinical efficacy of using these agents to treat truncal acne (Table 2).

### 8.1. Topical Management

Liu et al. [52] suggested that topical therapies could be the initial treatment options for mild-to-moderate truncal acne. Among a variety of topical agents used in acne, topical retinoids, benzoyl peroxide (BPO), azelaic acid, dapsone, and topical antibiotics can be used alone or in a combination of these for managing truncal acne.

BPO is a widely used topical agent for managing acne. BPO has a bactericidal efficacy against *C. acnes* and can reduce the inflammatory lesions of acne and the risk of antibiotic resistance. However, the previous use of BPO for treating the truncal area has been limited due to the bleaching effect of BPO on bed linens and clothing. Therefore, in the real-world, a wash-off cleanser formulation of BPO, including over-the-counter products, is commonly used for truncal acne. In 2010, Leyden et al. [53] designed a study examining the efficacy of 5.3% BPO emollient foam and 8% BPO wash in reducing *C. acnes* on the back of healthy individuals [53]. BPO 5.3% emollient foam decreased the total *C. acnes* count, whereas the 8% BPO wash did not significantly decrease *C. acnes* counts [53]. The authors suggested that the short contact time with the wash-off formulation was not enough for the sufficient penetration and deposition of BPO on the skin of the back. Therefore, the authors designed a further study which used a two-minute skin contact time with BPO wash. In that study, Leyden et al. [54] found that the application of 9.8% BPO foam to non-moistened skin during washing with a two-minute skin contact time in a once-daily application decreased the levels of *C. acnes* on the backs of healthy individuals. This suggests that the wash-off cleanser formulation of BPO could be used effectively for managing truncal acne.

Azelaic acid is a kind of natural compound that exerts its effect on acne through anti-keratinizing, anti-inflammatory, and antibacterial functions [55]. The topical application of azelaic acid is effective for both inflammatory acne and non-inflammatory acne lesions [55]. A prospective multicenter study in adult female patients with mild to moderate acne found that 12 weeks of treatment with twice-daily applications of 20% azelaic acid cream resulted in a significant improvement in acne on the face, chest, and back [56]. In addition, a significant decrease in the median dermatology life quality index (DLQI) was observed with very good or good tolerability of the treatment by both the patients and physicians [56]. A single center-pilot study found that 15% azelaic acid foam was effective in managing moderate truncal acne [57]. At week 16, 11 out of 16 patients with moderate truncal acne had clear or almost clear IGA scale scores after twice daily applications of 15% azelaic acid foam [57]. The authors suggested that this foam was effective in treating truncal acne due to its rapid skin penetration and spreadability [57]. A recent case series reported that the combination of 0.05% tretinoin lotion and 15% azelaic acid foam was effective in treating truncal acne in four female African-American patients [58].

The efficacy and safety of topical 7.5% dapsone gel were studied in patients with truncal acne [59]. Through 16 weeks of the treatment, 45% of the patients experienced clear/almost clear skin plus at least two-grade improvements in their truncal acne [59]. Topical 7.5% dapsone gel was relatively well-tolerated and safe for the patients throughout the study period [59].

Topical retinoids have been considered a potent treatment option for treatment of acne vulgaris. Several generations of topical retinoids have been developed. The binding affinity for several retinoid receptors and clinical considerations for use are summarized in Table 3. Among the diverse topical retinoids, Cunliffe et al. [60] reported that a 4-week treatment with topical isotretinoin was effective for facial and truncal acne without any clinically significant increase in plasma retinoid levels. A bioavailability study of 0.1% tazarotene foam compared to 0.1% tazarotene gel for moderate to severe acne was conducted [61]. Both agents were applied to the face, chest, shoulders, and upper back once daily. The authors concluded that both 0.1% tazarotene foam and 0.1% tazarotene gel were effective in managing moderate to severe acne with favorable safety and less potential for systemic exposure [61].

Recently, trifarotene, which selectively targets retinoic acid receptor gamma, was developed and shown to have potent anti-comedogenic, anti-inflammatory, and anti-pigmentary activities in an in vivo study [30]. The clinical effects of trifarotene on facial and truncal acne have been well demonstrated by clinical trials [62,63]. Two recent large-scale phase III clinical trials revealed that trifarotene cream was effective for both facial and truncal acne compared to the vehicle [62]. A long-term, open-label, 52-week study examining the safety and efficacy of trifarotene cream found that trifarotene was effective, safe, and well-tolerated by patients with moderate facial and truncal acne [63]. Of note, local irritation responses due to the application of trifarotene were less frequently observed in truncal acne than in facial acne [63], suggesting the tolerability of topical trifarotene in managing truncal acne.

The topical androgen receptor inhibitor clascoterone is a novel promising therapeutic option for acne. The mechanism of action of clascoterone on acne is supposed to be the inhibition of dihydrotestosterone-androgen receptor binding in sebocytes and the subsequent decrease in the downstream activation of androgen-driven lipid production and inflammation [64]. Two phase 3 clinical trials for clascoterone for facial acne were conducted from 2015 to 2018. The authors concluded that at week 12, 1% clascoterone cream significantly reduced the absolute noninflammatory lesion from baselines, with few and mild adverse events [65]. A nine-month, open-label extension trial to analyze the clinical safety and efficacy of clascoterone found that clascoterone could be used relatively safely and effectively for treating facial and truncal acne [64].

### 8.2. Systemic Management

Systemic management has been indicated for moderate to severe acne and inflammatory acne that are resistant to topical treatments. In moderate to severe acne, combined treatment with topical agents and systemic agents such as antibiotics, isotretinoin, oral contraceptives, or spironolactone can be applied [66].

The efficacy of tetracyclines and macrolides including tetracycline, doxycycline, minocycline, trimethoprim/sulfamethoxazole, trimethoprim, azithromycin, amoxicillin and cephalexin in treating acne has been well-documented [66]. Oral antibiotics are also effective options in managing truncal acne [67]. However, the use of trimethoprim-sulfamethoxazole and trimethoprim should be limited to patients who are unable to take tetracyclines or treatment-resistant patients. Systemic antibiotics should be used for the shortest possible duration of 3–4 months due to the potential for bacterial resistance [66]. Sarecycline is a third generation narrow-spectrum oral tetracycline that has been approved by the Food and Drug Administration (FDA) for treating acne [68]. Its unique structural characteristics enables it to exhibit anti-inflammatory and high anti-bacterial effects against *C. acnes*, *Staphylococci*, and *Streptococci* [69]. A phase 3 study of sarecycline for treating facial acne demonstrated that sarecycline was effective and well-tolerated [70]. A pooled analysis of two phase 3 studies on truncal acne was conducted [69]. Significant IGA success on the chest and back was, respectively, observed in the sarecycline group compared to the placebo group from three weeks to twelve weeks [69]. Thus, sarecycline could be a novel and effective option for managing moderate to severe truncal acne.

Isotretinoin is the treatment of choice for severe recalcitrant truncal acne [67]. The newer formulation of isotretinoin, isotretinoin-lidose, utilizes the lidose technique with micronized particles to increase the surface area of the drug. Therefore, isotretinoin-lidose can increase bioavailability by 50% compared to traditional isotretinoin. A double-blind, multicenter study of 925 patients with severe, recalcitrant nodular acne compared the clinical efficacy and safety of isotretinoin-lidose to standard isotretinoin for 20 weeks [71]. Both isotretinoin-lidose and traditional isotretinoin reduced the total number of nodules on the face and trunk to equivalent degrees, suggesting that both formulations had similar efficacy [71].

Hormone therapies such as oral contraceptives and spironolactone suppress the effect of androgens on the sebaceous glands, so they have been used for the treatment of acne.

Palli et al. [72] conducted a clinical trial to investigate the effects of 3 mg drospirenone/0.02 mg ethinyl estradiol on moderate truncal acne. Drospirenone has special antiandrogenic and anti-mineralocorticoid functions compared to other progestins. Due to these antiandrogenic properties, which can block male sex hormones associated with acne development, this hormone can be used to manage acne. In a single-center, double-blind, randomized study, 25 subjects, aged 18 to 45 years, with moderate truncal acne received 3 mg drospirenone/0.02 mg ethinyl estradiol or placebo for 24 weeks [72]. Treatment with 3 mg drospirenone/0.02 mg ethinyl estradiol showed treatment success among 53.3% of the patients based on IGA, and 60% of the patients based on subject global assessment (SGA) [72]. This regimen demonstrated significant clinical improvement with good tolerability by the patients [72].

### 8.3. Procedural Therapies

In general, truncal acne tends to have a slower response to treatment than facial acne, so it is likely to be approached with a more multidisciplinary treatment strategy. Among the variety of procedural therapies that can be applied to acne lesions such as lasers, light devices, chemical peels, and intralesional injections, there is a conspicuous absence of many randomized controlled trials evaluating the efficacy of these modalities in treating truncal acne. Regarding chemical peels, salicylic acid, glycolic acid, lactic acid, mandelic acid, retinoic acid, trichloroacetic acid, Jessner’s solution, kojic acid, pyruvic acid, azelaic acid, and combination peels are used in management of acne [73]. Although most of the studies on chemical peels for acne studied facial acne, in general practice, most of the chemical peels are also applied to the trunk [73]. Among them, photodynamic therapy (PDT) has the greatest evidence for the management of truncal acne. A meta-analysis reported that PDT was effective in improving inflammatory acne vulgaris based on the analysis of 13 randomized controlled trials [74]. For truncal acne, the clinical efficacy of PDT with topical 5% 5-aminolevulinic acid was evaluated in Asian patients with truncal acne [75]. With a single treatment session with 5% 5-aminolevulinic acid PDT, a 64.2% reduction in inflammatory lesion counts and a 24.3% reduction in non-inflammatory lesion counts of truncal acne were observed at 12 weeks of follow-up among 15 patients [75].

For managing hypertrophic truncal scars, intralesional injections with corticosteroids, 5-fluorouracil, or bleomycin can be performed. In addition, cryotherapy and PDT can also be promising treatment modalities for managing hypertrophic truncal acne scars.

### 8.4. Future Directions

Although there is little clinical evidence on treatments to manage truncal acne, truncal acne is generally controlled in a similar way to that of facial acne. In the case of topical or oral formulations that have been recently developed, several clinical reports presented the effect of those agents in treating truncal acne. Although truncal acne occurs in covered areas, hypertrophic scars are common, calling for vigorous treatment as a scar preventive measure. Therefore, awareness should be established to actively treat body acne at an early stage.

## 9. Conclusions

Truncal acne is a condition that, despite being relatively common, is underreported by patients and underdiagnosed and undertreated by clinicians. More research on the epidemiology of truncal acne is needed, and in relation to treatment, evidence for effective therapeutic agents for truncal acne needs to be established independently of facial acne. Truncal acne can cause persistent scarring, such as macular atrophic scarring and keloids. Therefore, the early and prompt treatment of truncal acne patients is needed to prevent further scarring. In truncal acne patients who already have scars due to previous truncal acne, more attention and aggressive treatment should be conducted to normalize the condition.

Thus, there is an unmet need for treating truncal acne. As truncal acne occurs on an unexposed body part, more effort by the clinician to examine the lesions in detail is needed. In addition to multidisciplinary research for trunk acne to reveal its pathogenic characteristics and proper management options, significant effort should also be put into increasing the awareness of the importance of early and appropriate treatment for truncal acne.

## Figures and Tables

**Table 1:** Summary of recently developed assessment tool for truncal acne.

Tool	Sites	Grading	Descriptions of the Grading
LRAG	Each of face, chest, and back	Based on photographic templates Based on the number of inflamed lesions and their inflammatory intensity (Grade 1–12) Predominant noninflamed lesions (Grade 1–3)	Grade 1, least severe–Grade 12, most severe Grade 1, least severe–Grade 3, most severe
CLASS	Each of face, chest, and back	Clear (0) Almost clear (1) Mild (2) Moderate (3) Severe (4) Very severe (5)	No lesions to barely noticeable ones, very few scattered comedones and papules Hardly visible from 2.5 m away. A few scattered comedones, few small papules, and very few pustules Easily recognizable; less than half of the affected area is involved. Many comedones, papules, and pustules; 0–5 papules and pustules More than half of the affected area is involved; numerous comedones, papules and pustules; 6–20 papules and pustules The entire area is involved. Covered with comedones, numerous pustules, and papules, a few nodules and cyst; 21–50 papules and pustules Highly inflammatory acne covering the affected area, with nodules and cyst present; >50 inflammatory lesions
SCAR-S	Each of face, chest, and back	The overall scar score is the sum of scores from each of these three sites. Clear (0) Almost clear (1) Mild (2) Moderate (3) Severe (4) Very severe (5)	No visible scars from acne Hardly visible scars from 2.5 m away Easily recognizable; less than half the affected area (e.g., face, back, or chest) involved More than half the affected area (e.g., face, back, or chest) involved Entire area involved Entire area with prominent atrophic or hypertrophic scars
TRASS	Trunk (upper back, lower back, and chest)	Sum of the three sub-score items (ranging from 0–19) Sub-score 1: criteria of severity based on disease and family history (maximum score 6) Sub-score 2: Clinical marker of acne severity (maximum score 11) Sub-score 3: quality of life (maximum score 2)	Item 1: Duration of acne (0, 0–2 years; 1, 3–5 years; 2, ≥6 years) Item 2: Family history (0, none; 1, present) Item 3: Past systemic acne treatment (0, none; 1, oral systemics; 2, isotretinoin) Item 4: Areas on the trunk Upper back (0, no lesion; 1, lesion) Lower back (0, no lesion; 1, lesion) Chest (0, no lesion; 1, lesion) Item 5: Number of nodules (0, 0; 1, 1–5; 2, 6–9; 3, ≥10) Item 6: Scars (Hypertrophic/keloid: 0, no; 1, yes; atrophic: 0, no; 1, yes; elastosis: 0, no; 1, yes; hyperpigmentation/erythema: 0, no; 1, yes) Item 7: Facial acne (0, no; 1, yes) Item 8: Severity of impact on quality of life (0, none; 1, moderate; 2, important)

Abbreviation: CLASS, the comprehensive acne severity scale; LRAG, Leeds Revised Acne grading; SCAR-S, six-category global severity scale; TRASS, truncal acne severity scale.

**Table 2:** Summary of clinical trials for management of truncal acne.

Drug (Trade Name)	Company	Clinical Trial Number	Phase and Status	Study Design	Results
*Topical management*					
Dapsone 7.5 % gel (Aczone^®^)	Almirall	NCT02944461	Phase 4, completed	A 16-week open label pilot study (*n* = 20) with moderate truncal acne	At week 16, 46.7% showed at least two grade improvement and a rating of clear or almost clear on the investigator global assessment (IGA) scale.
Azelaic acid 20% cream (Skinoren^®^)	GSK	N/A	Phase 4, completed	A prospective, noninterventional multicenter study in adult female patients (*n* = 251) with mild to moderate acne	A significant improvement of acne on the face, chest, and back was observed after 12 weeks of the treatment.
Tazarotene foam and gel (Tazorac^®^)	GSK	NCT01019603	Phase 1, completed	A single-center, randomized, open-label, comparative bioavailability study in subjects with moderate to severe acne vulgaris (*n* = 30)	Mean concentration of tazarotene was higher for gel versus foam.
Trifarotene (Aklief^®^)	Galderma	NCT02189629 NCT02566369 NCT02556788	Phase 3, completed Phase 3, completed	An open-label, 52-week study with moderate facial and truncal acne (*n* = 453) Two double-blind, randomized, vehicle-controlled, 12-weeks studies of trifarotene cream versus vehicle in moderate facial and truncal acne	66.9% patients with truncal acne demonstrated treatment success by physician’s global assessment rating (no or almost no acne). A highly significant difference in favor of trifarotene compared to the vehicle was observed based on physician’s global assessment and the change in inflammatory and noninflammatory lesion counts.
Cortexolone 17a-propionate (CB-03-01) 1% cream (Clascoterone^®^)	Cassiopea S.p.A.	NCT02682264	Phase 3, completed	An open-label, long-term extension study (*n* = 609) to evaluate safety with acne	The 1% clascoterone cream showed a well-tolerable safety profile.
*Systemic management*					
Sarecycline (Seysara^®^)	Almirall	NCT02322866 NCT05010538	Phase 3, completed Phase 4, Active, not recruiting	Two double-blind, randomized, 12-week studies of sarecycline in the treatment of acne A 12-week, single center open-label case series study for truncal acne (*n* = 10)	The pooled analysis for truncal acne showed that chest and back IGA success rate was significantly greater with sarecycline than with the placebo at week 3, 6, and 12, respectively.
Isotretinoin-Lidose (Absorica^®^)	Galephar	N/A	Phase 3, completed	A 20-week, multicenter, double-blind randomized study to evaluate the safety and efficacy of isotretinoin-lidose in patients with severe recalcitrant nodular facial and truncal acne (*n* = 925)	The mean change in facial and truncal nodular lesion counts from week 0 to week 20 was comparable between the isotretinoin-lidose group and the food-dependent generic isotretinoin group.
Drospirenone and Ethinyl Estradiol (YAZ^®^)	Bayer	NCT00722761	Phase 3, completed	A single center, randomized double-blind, parallel group study in moderate truncal acne vulgaris (*n* = 30)	The drospirenone/ethinyl estradiol showed treatment success among 53.3% of the patients based on IGA and 60% of the patients based on subject global assessment.

Abbreviation: N/A, not accessible.

**Table 3:** Summary of topical retinoids for acne.

Retinoids	Trade Name (Formulations)	Binding Affinity to RARs	US FDA Pregnancy Category	Half-Life in Plasma
All-trans retinoic acid (Tretinoin)	Stieva-A^®^, Atralin^®,^ Avita^®^, Retin-A^®,^ Retin-A Micro^®^, Tretin-X^®^ (Cream: 0.01%, 0.025%, 0.05%; gel: 0.01%, 0.025%, 0.04%, 0.05%, 0.1%)	RARα(++), RARβ(++), RARγ(++)	Category C	0.5–2 h
Adapalene	Differin^®^ (Cream: 0.1%; gel: 0.1%, 0.3%; lotion: 0.1%)	RARβ(++), RARγ(++)	Category C	7–51 h
Tazarotene	Tazorac^®^ (Cream: 0.05%, 0.1%; gel: 0.05%, 0.1%; foam: 0.1%)	RARα(+), RARβ(+++), RARγ(++)	Category X	18 h
Trifarotene	AKLIEF^®^ (Cream: 0.005%)	RARγ(+++)	Not assigned	2–9 h

(+), Minimal binding affinity; (++) moderate binding affinity; (+++) strong binding affinity; Abbreviations: FDA, food and drug administration; RAR, retinoic acid receptor.

## References

[1] Vos T., Flaxman A.D., Naghavi M., Lozano R., Michaud C., Ezzati M., Shibuya K., Salomon J.A., Abdalla S., Aboyans V. (2012). Years lived with disability (YLDs) for 1160 sequelae of 289 diseases and injuries 1990–2010: A systematic analysis for the Global Burden of Disease Study 2010. Lancet.

[2] Heng A.H.S., Chew F.T. (2020). Systematic review of the epidemiology of acne vulgaris. Sci. Rep..

[3] Marson J.W., Baldwin H.E. (2021). Isotretinoin update. Dermatol. Rev..

[4] Del Rosso J.Q., Bikowski J.B., Baum E., Smith J., Hawkes S., Benes V., Bhatia N. (2007). A closer look at truncal acne vulgaris: Prevalence, severity, and clinical significance. J. Drugs Dermatol. JDD.

[5] Isaacsson V.C.S., Almeida H.L.D., Duquia R.P., Breunig J.D.A., Souza P.R.M.D. (2014). Dissatisfaction and acne vulgaris in male adolescents and associated factors. An. Bras. De Dermatol..

[6] Dreno B., Thiboutot D., Layton A., Berson D., Perez M., Kang S. (2015). on behalf of the Global Alliance to Improve Outcomes in Acne. Large-scale international study enhances understanding of an emerging acne population: Adult females. J. Eur. Acad. Dermatol. Venereol..

[7] Dréno B., Jean-Decoster C., Georgescu V. (2016). Profile of patients with mild-to-moderate acne in Europe: A survey. Eur. J. Dermatol..

[8] Barth J.H., Clark S. (2003). Acne and hirsuties in teenagers. Best Pract. Res. Clin. Obstet. Gynaecol..

[9] Poli F., Auffret N., Leccia M.T., Claudel J.P., Dréno B. (2020). Truncal acne, what do we know?. J. Eur. Acad. Dermatol. Venereol..

[10] Nijsten T., Rombouts S., Lambert J. (2007). Acne is prevalent but use of its treatments is infrequent among adolescents from the general population. J. Eur. Acad. Dermatol. Venereol..

[11] Wei B., Pang Y., Zhu H., Qu L., Xiao T., Wei H.C., Chen H.D., He C.D. (2010). The epidemiology of adolescent acne in North East China. J. Eur. Acad. Dermatol. Venereol..

[12] Suh D.H., Kim B.Y., Min S.U., Lee D.H., Yoon M.Y., Kim N.I., Kye Y.C., Lee E.S., Ro Y.S., Kim K.J. (2011). A multicenter epidemiological study of acne vulgaris in Korea. Int. J. Dermatol..

[13] Dessinioti C., Katsambas A. (2017). Difficult and rare forms of acne. Clin. Dermatol..

[14] Zaba R., Schwartz R., Jarmuda S., Czarnecka–Operacz M., Silny W. (2011). Acne fulminans: Explosive systemic form of acne. J. Eur. Acad. Dermatol. Venereol..

[15] Gollnick H. (2015). From new findings in acne pathogenesis to new approaches in treatment. J. Eur. Acad. Dermatol. Venereol..

[16] Zouboulis C., Eady A., Philpott M., Goldsmith L., Orfanos C., Cunliffe W., Rosenfield R. (2005). What is the pathogenesis of acne?. Exp. Dermatol..

[17] Kim B.R., Chun M.Y., Kim S.A., Youn S.W. (2015). Sebum secretion of the trunk and the development of truncal acne in women: Do truncal acne and sebum affect each other?. Dermatology.

[18] Melnik B., Jansen T., Grabbe S. (2007). Abuse of anabolic-androgenic steroids and bodybuilding acne: An underestimated health problem. JDDG J. Der Dtsch. Dermatol. Ges..

[19] Zouboulis C.C. (2020). Endocrinology and immunology of acne: Two sides of the same coin. Exp. Dermatol..

[20] Deng Y., Wang H., Zhou J., Mou Y., Wang G., Xiong X. (2018). Patients with Acne Vulgaris Have a Distinct Gut Microbiota in Comparison with Healthy Controls. Acta Derm. Venereol..

[21] Yan H.M., Zhao H.J., Guo D.Y., Zhu P.Q., Zhang C.L., Jiang W. (2018). Gut microbiota alterations in moderate to severe acne vulgaris patients. J. Dermatol..

[22] Huang Y., Liu L., Chen L., Zhou L., Xiong X., Deng Y. (2021). Gender-Specific Differences in Gut Microbiota Composition Associated with Microbial Metabolites for Patients with Acne Vulgaris. Ann. Dermatol..

[23] Dagnelie M.A., Montassier E., Khammari A., Mounier C., Corvec S., Dréno B. (2019). Inflammatory skin is associated with changes in the skin microbiota composition on the back of severe acne patients. Exp. Dermatol..

[24] Dagnelie M.-A., Corvec S., Saint-Jean M., Bourdès V., Nguyen J.-M., Khammari A., Dréno B. (2018). Decrease in diversity of Propionibacterium acnes phylotypes in patients with severe acne on the back. Acta Derm. Venereol..

[25] Kim J., Park T., Kim H.-J., An S., Sul W.J. (2021). Inferences in microbial structural signatures of acne microbiome and mycobiome. J. Microbiol..

[26] Nguyen H.L., Tollefson M.M. (2017). Endocrine disorders and hormonal therapy for adolescent acne. Curr. Opin. Pediatrics.

[27] Lolis M.S., Bowe W.P., Shalita A.R. (2009). Acne and systemic disease. Med. Clin..

[28] Bansal P., Sardana K., Sharma L., Garga U.C., Vats G. (2021). A prospective study examining isolated acne and acne with hyperandrogenic signs in adult females. J. Dermatol. Treat..

[29] Saint-Jean M., Khammari A., Jasson F., Nguyen J.-M., Dréno B. (2016). Different cutaneous innate immunity profiles in acne patients with and without atrophic scars. Eur. J. Dermatol..

[30] Aubert J., Piwnica D., Bertino B., Blanchet-Réthoré S., Carlavan I., Déret S., Dreno B., Gamboa B., Jomard A., Luzy A. (2018). Nonclinical and human pharmacology of the potent and selective topical retinoic acid receptor-γ agonist trifarotene. Br. J. Dermatol..

[31] Kim S.A., Kim B.R., Chun M.Y., Youn S.W. (2016). Relation between pH in the trunk and face: Truncal pH can be easily predicted from facial pH. Ann. Dermatol..

[32] Pillsbury D.M., Shelley W.B. (1954). Dermatology. Annu. Rev. Med..

[33] Burke B.M., Cunliffe W. (1984). The assessment of acne vulgaris—The Leeds technique. Br. J. Dermatol..

[34] O’brien S., Lewis J., Cunliffe W. (1998). The Leeds revised acne grading system. J. Dermatol. Treat..

[35] Tan J.K., Tang J., Fung K., Gupta A.K., Thomas D.R., Sapra S., Lynde C., Poulin Y., Gulliver W., Sebaldt R.J. (2007). Development and validation of a comprehensive acne severity scale. J. Cutan. Med. Surg..

[36] Tan J.K., Tang J., Fung K., Gupta A.K., Thomas D.R., Sapra S., Lynde C., Poulin Y., Gulliver W., Sebaldt R.J. (2010). Development and validation of a Scale for Acne Scar Severity (SCAR-S) of the face and trunk. J. Cutan. Med. Surg..

[37] Bernardis E., Shou H., Barbieri J.S., McMahon P.J., Perman M.J., Rola L.A., Streicher J.L., Treat J.R., Castelo-Soccio L., Yan A.C. (2020). Development and initial validation of a multidimensional acne global grading system integrating primary lesions and secondary changes. JAMA Dermatol..

[38] Auffret N., Nguyen J., Leccia M.T., Claudel J., Dréno B. (2022). TRASS: A global approach to assess the severity of truncal acne. J. Eur. Acad. Dermatol. Venereol..

[39] Tan J., Alexis A., Baldwin H., Beissert S., Bettoli V., Del Rosso J., Dréno B., Gold L.S., Harper J., Lynde C. (2021). Gaps and recommendations for clinical management of truncal acne from the Personalising Acne: Consensus of Experts panel. JAAD Int..

[40] Sun K.-L., Chang J.-M. (2017). Special types of folliculitis which should be differentiated from acne. Derm. Endocrinol..

[41] Prindaville B., Belazarian L., Levin N.A., Wiss K. (2018). Pityrosporum folliculitis: A retrospective review of 110 cases. J. Am. Acad. Dermatol..

[42] Rubenstein R.M., Malerich S.A. (2014). Malassezia (pityrosporum) folliculitis. J. Clin. Aesthetic Dermatol..

[43] Kazandjieva J., Tsankov N. (2017). Drug-induced acne. Clin. Dermatol..

[44] Mallon E., Newton J., Klassen A., Stewart-Brown S.L., Ryan T., Finlay A. (1999). The quality of life in acne: A comparison with general medical conditions using generic questionnaires. Br. J. Dermatol..

[45] Rocha M.A., Bagatin E. (2018). Adult-onset acne: Prevalence, impact, and management challenges. Clin. Cosmet. Investig. Dermatol..

[46] Papadopoulos L., Walker C., Aitken D., Bor R. (2000). The relationship between body location and psychological morbidity in individuals with acne vulgaris. Psychol. Health Med..

[47] Tan J., Beissert S., Cook-Bolden F., Chavda R., Harper J., Hebert A., Lain E., Layton A., Rocha M., Weiss J. (2021). Impact of facial and truncal acne on quality of life: A multi-country population-based survey. JAAD Int..

[48] Layton A., McDonald B., Mohd Mustapa M., Levell N. (2022). National Institute for Health and Care Excellence (NICE) acne guideline: What’s the latest for dermatologists?. Br. J. Dermatol..

[49] Xu J., Mavranezouli I., Kuznetsov L., Murphy M.S., Healy E. (2021). Management of acne vulgaris: Summary of NICE guidance. BMJ.

[50] Harper J.C. (2004). An update on the pathogenesis and management of acne vulgaris. J. Am. Acad. Dermatol..

[51] Conforti C., Chello C., Giuffrida R., di Meo N., Zalaudek I., Dianzani C. (2020). An overview of treatment options for mild-to-moderate acne based on American Academy of Dermatology, European Academy of Dermatology and Venereology, and Italian Society of Dermatology and Venereology guidelines. Dermatol. Ther..

[52] Liu C., Tan J. Understanding truncal acne: A practical guide to diagnosis and management. Skin Ther. Lett..

[53] Leyden J.J. (2010). Efficacy of benzoyl peroxide (5.3%) emollient foam and benzoyl peroxide (8%) wash in reducing Propionibacterium acnes on the back. J. Drugs Dermatol. JDD.

[54] Leyden J.J., Del Rosso J.Q. (2012). The effect of benzoyl peroxide 9.8% emollient foam on reduction of Propionibacterium acnes on the back using a short contact therapy approach. J. Drugs Dermatol. JDD.

[55] Shemer A., Weiss G., Amichai B., Kaplan B., Trau H. (2002). Azelaic acid (20%) cream in the treatment of acne vulgaris. J. Eur. Acad. Dermatol. Venereol..

[56] Kainz J.T., Berghammer G., Auer-Grumbach P., Lackner V., Perl-Convalexius S., Popa R., Wolfesberger B. (2016). Azelaic acid 20% cream: Effects on quality of life and disease severity in adult female acne patients. JDDG J. Der Dtsch. Dermatol. Ges..

[57] Hoffman L.K., Del Rosso J.Q., Kircik L.H. (2017). The Efficacy and Safety of Azelaic Acid 15% Foam in the Treatment of Truncal Acne Vulgaris. J. Drugs Dermatol. JDD.

[58] Surin-Lord S.S., Miller J. (2020). Topical treatment of truncal acne with tretinoin lotion 0.05% and azelaic acid foam. Case Rep. Dermatol. Med..

[59] Del Rosso J.Q., Kircik L., Tanghetti E. (2018). Management of truncal acne vulgaris with topical dapsone 7.5% gel. J. Clin. Aesthetic Dermatol..

[60] Cunliffe W.J., Glass D., Goode K., Stables G.I., Boorman G.C. (2001). A double-blind investigation of the potential systemic absorption of isotretinoin, when combined with chemical sunscreens, following topical application to patients with widespread acne of the face and trunk. Acta Derm. Venereol..

[61] Jarratt M., Werner C.P., Alió Saenz A.B. (2013). Tazarotene foam versus tazarotene gel: A randomized relative bioavailability study in acne vulgaris. Clin. Drug Investig..

[62] Tan J., Thiboutot D., Popp G., Gooderham M., Lynde C., Del Rosso J., Weiss J., Blume-Peytavi U., Weglovska J., Johnson S. (2019). Randomized phase 3 evaluation of trifarotene 50 μg/g cream treatment of moderate facial and truncal acne. J. Am. Acad. Dermatol..

[63] Blume-Peytavi U., Fowler J., Kemény L., Draelos Z., Cook-Bolden F., Dirschka T., Eichenfield L., Graeber M., Ahmad F., Saenz A.A. (2020). Long-term safety and efficacy of trifarotene 50 μg/g cream, a first-in-class RAR-γ selective topical retinoid, in patients with moderate facial and truncal acne. J. Eur. Acad. Dermatol. Venereol..

[64] Eichenfield L., Hebert A., Gold L.S., Cartwright M., Fragasso E., Moro L., Mazzetti A. (2020). Open-label, long-term extension study to evaluate the safety of clascoterone (CB-03-01) cream, 1% twice daily, in patients with acne vulgaris. J. Am. Acad. Dermatol..

[65] Hebert A., Thiboutot D., Gold L.S., Cartwright M., Gerloni M., Fragasso E., Mazzetti A. (2020). Efficacy and safety of topical clascoterone cream, 1%, for treatment in patients with facial acne: Two phase 3 randomized clinical trials. JAMA Dermatol..

[66] Zaenglein A.L., Pathy A.L., Schlosser B.J., Alikhan A., Baldwin H.E., Berson D.S., Bowe W.P., Graber E.M., Harper J.C., Kang S. (2016). Guidelines of care for the management of acne vulgaris. J. Am. Acad. Dermatol..

[67] Del Rosso J.Q., Stein-Gold L., Lynde C., Tanghetti E., Alexis A.F. (2019). Truncal acne: A neglected entity. J. Drugs Dermatol. JDD.

[68] Del Rosso J.Q. (2020). Sarecycline and the Narrow-spectrum tetracycline concept: Currently Available Data and Potential Clinical Relevance in Dermatology. J. Clin. Aesthetic Dermatol..

[69] Del Rosso J.Q., Baldwin H., Harper J., Zeichner J., Obagi S., Graber E., Jimenez X., Vicente F., Grada A. (2021). Management of Truncal Acne With Oral Sarecycline: Pooled Results from Two Phase-3 Clinical Trials. J. Drugs Dermatol. JDD.

[70] Moore A., Green L.J., Bruce S., Sadick N., Tschen E., Werschler P., Cook-Bolden F.E., Dhawan S.S., Forsha D., Gold M.H. (2018). Once-Daily Oral Sarecycline 1.5 mg/kg/day Is Effective for Moderate to Severe Acne Vulgaris: Results from Two Identically Designed, Phase 3, Randomized, Double-Blind Clinical Trials. J. Drugs Dermatol. JDD.

[71] Webster G.F., Leyden J.J., Gross J.A. (2014). Results of a Phase III, double-blind, randomized, parallel-group, non-inferiority study evaluating the safety and efficacy of isotretinoin-Lidose in patients with severe recalcitrant nodular acne. J. Drugs Dermatol. JDD.

[72] Palli M., Reyes-Habito C.M., Lima X.T., Kimball A.B. (2013). A single-center, randomized double-blind, parallel-group study to examine the safety and efficacy of 3mg drospirenone/0.02 mg ethinyl estradiol compared with placebo in the treatment of moderate truncal acne vulgaris. J. Drugs Dermatol. JDD.

[73] Castillo D.E., Keri J.E. (2018). Chemical peels in the treatment of acne: Patient selection and perspectives. Clin. Cosmet. Investig. Dermatol..

[74] Tang X., Li C., Ge S., Chen Z., Lu L. (2020). Efficacy of photodynamic therapy for the treatment of inflammatory acne vulgaris: A systematic review and meta-analysis. J. Cosmet. Dermatol..

[75] Yew Y.W., Lai Y.C., Lim Y.L., Chong W.-S., Theng C. (2016). Photodynamic Therapy With Topical 5% 5-Aminolevulinic Acid for the Treatment of Truncal Acne in Asian Patients. J. Drugs Dermatol. JDD.

